# Bletilla oligosaccharides improved 5-fluorouracil-induced intestinal mucositis in mice by activating NF-κB signalling pathway and regulating intestinal microbiota

**DOI:** 10.3389/fphar.2025.1526274

**Published:** 2025-03-13

**Authors:** Qiuxiong Yin, Xinran Li, Yanli Xiong, Yupeng Jiang, Shengsuo Ma, Guoqiang Qian

**Affiliations:** ^1^ School of Traditional Chinese Medicine, Guangdong Pharmaceutical University, Guangzhou, China; ^2^ Department of Experimental Research, Sun Yat-sen University Cancer Center, Guangzhou, China

**Keywords:** Bletilla oligosaccharides, chemotherapeutic intestinal mucositis, inflammation, intestinal flora, NF-kB

## Abstract

**Introduction:**

The Bletilla oligosaccharides (BO) are active compounds extracted from *Bletilla striata* and have the strong protective effect on the gastrointestinal tract. Chemotherapeutic intestinal mucositis (CIM) is one of the toxic side effects of chemotherapeutic agents on the gastrointestinal tract. The aim of this study was to identify the structure of BO and evaluate the therapeutic effect of BO on 5-fluorouracil-induced intestinal mucosal inflammation.

**Methods:**

BO were purified from DEAE52 cellulose. The structure of BO were characterised by HPGPC, GC-MS and NMR. *In vivo*, the mouse model of intestinal mucositis was established by intraperitoneal injection of 5-FU. The effect of BO on intestinal mucositis in mice was detected by assessing the levels of intestinal flora, ZO-1, occludin, and MUC-2, and inflammatory cytokines (IL-1β, IL-6, IL-10, and TNF-α).

**Results:**

Structural characterisation showed that BO were the neutral polysaccharide composed mainly of glucose and mannose. The backbone of BO consisted of→4)-β-Manp-(1→, →4)-β-Glcp-(1→ and small →3,4)-α-Manp-(1→. The results of the *in vivo* experiment showed that the symptoms of diarrhoea, haematochezia and colonic mucosal lesions improved after administration of BO. Further experiments showed that BO not only reduced the levels of pro-inflammatory factors such as IL-1β, IL-6 and TNF-α, but also improved the expression of intestinal barrier protein and intestinal microbial community after BO treatment.

**Conclusion:**

BO can relieve the progress of intestinal mucositis by relieving inflammation, protecting the intestinal epithelial barrier and regulating the intestinal microbiota. These data provide experimental evidence for the application of BO in chemotherapeutic intestinal mucositis.

## 1 Introduction

A popular antimetabolic chemotherapy drug, 5-fluorouracil (5-FU) is used to treat a variety of cancers, including skin, stomach, bladder, colorectal, breast, and rectal tumors (Mafi, et al., 2023). 5-FU inhibits the growth and division of fast-growing cells, thereby killing them. However, the specificity of chemotherapy drugs are limited and fast-growing healthy cells can also be attacked and damaged. The non-specific effects of 5-FU can lead to a number of side effects (Cinausero, et al., 2017; Soveri, et al., 2014). Among these, chemotherapy intestinal mucositis (CIM) is one of the most serious side effects of 5-FU therapy, which has a significant negative impact on the clinical treatment of patients and can even lead to death in the event of severe complications (Al-Dasooqi, et al., 2013). It is also the main cause of reduced survival and early death (Ribeiro, et al., 2016; Sougiannis, et al., 2021). However, as the pathological mechanism of 5-FU-induced intestinal mucositis is still controversial, there is currently no preventive strategy or appropriate treatment for intestinal mucositis, and treatment is mainly focused on relieving the symptoms of mucositis (Van Sebille, et al., 2015). The existing clinical drugs have limited efficacy and certain side effects. Therefore, it is urgent to take effective measures to control intestinal mucositis and improve the quality of patients’ life.

*Bletilla striata* is a species of plant in the Orchidaceae family, mainly found in southern and eastern China near the Yangtze River, Japan, Korea, Vietnam and other places (Zhang, et al., 2022a). In China, *Bletilla striata* has been defined as a medicine food homology species due to its nutritional and medicinal ingredients. According to the theory of traditional Chinese medicine, *Bletilla striata* has an astringent, haemostatic, decongestant and muscle-building effect (Jiang, et al., 2021). The diverse effects of *Bletilla striata* are attributed to its various active compounds (Xu, et al., 2019), of which the polysaccharides have gradually attracted attention and are considered the most important active compounds in *Bletilla striata*. In recent decades, the polysaccharides of *Bletilla striata* have broad development potential as functional food supplements, therapeutic agents and biological materials. *Bletilla striata polysaccharides* (BSP) have immunomodulatory (Wang et al., 2019), antioxidant (Chen et al., 2021), gastroprotective (Wang et al., 2020), anti-inflammatory (Wang et al., 2020) and antitumour (Liu et al., 2022) effects. There is increasing evidence that BSP has a protective effect on the intestinal epithelium. They can inhibit the levels of the inflammatory cytokines IL-6 and TNF-α, increase the expression of the intestinal epithelial tight junction protein and can be used as a new protective agent to restore the normal function of the intestinal epithelial barrier (Luo, et al., 2019). The biological function of BSP is largely determined by its structural characteristics, including monosaccharide composition, molecular weight, type of glycosyl bonds, etc. The molecular weight of BSP ranges from 12.6 to 820 kDa (Zhu et al., 2023). Due to the diversity and complexity of its structure, the activity and application of BSP are limited. Therefore, in this study, by hydrolyzing BSP with trifluoroacetic acid, Bletilla oligosaccharides with lower molecular weight and viscosity were produced. Oligosaccharides are low molecular weight polymers consisting of 2–10 monosaccharides linked by glycosidic bonds. By reducing the molecular weight and simplifying the structure, their solubility, stability and safety were improved (Liu M. et al., 2021). BO have been confirmed to alleviate DSS-induced colon injury (Zhu et al., 2022), but its protective effect on chemotherapeutic agent 5-FU-induced intestinal mucositis needs further investigation.

Therefore, in this study, we proposed to effectively degrade BSP into oligosaccharides, identify and characterise its structure. Subsequently, we aimed to determine the role of BO in the inhibition of CIM in 5-FU-treated mice and further investigate whether the gut microbiota can influence this response.

## 2 Materials and methods

### 2.1 Materials

*Bletilla striata* was purchased from Beijing Tongrentang Guangzhou Pharmaceutical chain Co., LTD. DEAE-52 cellulose was obtained from Beijing Solarbio Science & Technology Co.,Ltd. (Beijing, China. Lot:C8930-100 g). 5-FU (PubChem CID: 3385) was purchased from Sigma-Aldrich Co. LLC (Shanghai, China. Lot:900394-1g). Antibodies of phospho-NF-κB p65 (Lot:82335-1-RR), MyD88 (Lot:29946-1-AP), TLR4 (Lot:19811-1-AP) and β-actin (Lot: 20536-1-AP) were obtained from proteintech, USA. All organic solvents used in the analysis were kept in chromatographic purity, while the other reagents were kept in analytical purity.

### 2.2 Preparation of BO

100.0 g *Bletilla striata* powder was added to 1,000 mL pure water, soaked overnight in 37°C, then mixtures were centrifuged (1,000 rpm, 3 min), the residue was extracted twice again. The supernatant was collected and concentrated by a rotary evaporator under a vacuum. To precipitate the polysaccharides, added anhydrous ethanol, then placed at 4°C overnight. Following the preparation of *B. striata polysaccharide* in 1% sugar solution, 1.25M trifluoroacetic acid (TFA) was added at a 1:1 (v/v) ratio, and kept at 95°C for 2 hours to obtain BO. Then, the remaining TFA in BO was removed by vacuum rotary steaming. After being chromatographically segregated using a DEAE-cellulose ion exchange column, eluted with distilled water and NaCl solution, and freeze-dried, the water-washed neutral polysaccharides fraction was eventually obtained. After the salt ions were eliminated using a dialysis bag, High Performence Gel Permeation Chromatography (HPGPC), Nuclear Magnetic Resonance Spectroscopy (NMR), Mass spectrometry - Gas chromatography (GC-MS) were used to assess the fractionated polysaccharide fractions.

### 2.3 Determination of Mw

The chromatographic system was a gel chromatograph-differential multi-angle laser light scattering system. The liquid phase system was U3000 (Thermo, USA), and the differential detector was Optilab T-rEX (Wyatt technology, CA, USA). The laser light scattering detector is DAWN HELEOS II (Wyatt technology, CA, USA). The sample was dissolved in 0.1M NaNO_3_ aqueous solution (with 0.02% NaN_3_, w/w) with a final concentration of 1 mg/mL and filtered through a philtre with a pore size of 0.45 μm. The injection volume was 100 μL and the column temperature was 45°C. The eluent was an aqueous solution containing 0.1M NaNO_2_ and 0.02% NaN_3_, and the isometric elution took 75 min. The flow rate was 0.6 mL/min.

### 2.4 Monosaccharide composition

Take a clean chromatography vial, weigh out an appropriate amount of polysaccharide samples, add 1 mL of 2M TFA acid solution and heat to 121°C for 2 h. After running the nitrogen, blow dry. Clean the samples with 99.99% methanol and then blow dry. Repeat the methanol cleaning 2–3 times. Dissolve them in sterile water and transfer them to the chromatography vial for measurement. Thermo ICS 5000+ ion chromatography system (ICS 5000+, Thermo Fisher Scientific, USA) was used in the chromatographic system. It analyzed and detected the monosaccharide components using an electrochemical detector.

### 2.5 Methylation analysis

The procedure involved dissolving 2 mg of Bletilla oligosaccharides in 500 μL of DMSO, then adding 1 mg of NaOH and 50 μL of iodomethane solution for an hour. Next, 1 mL of water and 2 mL of dichloromethane were added, mixed in a vortex, centrifuged, and the water phase was discarded after three repeated washes. Finally, the lower dichloromethane phase was absorbed and dried with nitrogen. Next, mix in 50 μL of 2 M ammonia and 50 μL of 1 M NaBD_4_, let it react for 2.5 h at room temperature, then put 20 μL of acetic acid in to halt it, and finally dry it with nitrogen. Afterwards, 250 μL of acetic anhydride was added, followed by a vortex, mixing, and 2.5 h of reaction at 100°C. 500 μL of dichloromethane was then added, and the lower dichloromethane phase was removed and identified using GC-MS. Agilent gas chromatography system (Agilent 7890A; Agilent Technologies, USA) with column BPX70 (30 m × 0.25 mm × 0.25 µm, SGE, Australia) was the chromatographic system used. High-purity helium was used as the carrier gas, the sample size was 1 μL, and the shunt ratio was 10:1. After being at 140°C for 2 minutes, the column temperature chamber was heated to 230°C in 3 minutes at a rate of 3°C per minute.

### 2.6 NMR analysis

BO was completely dissolved in D_2_O to yield a polysaccharide solution with a concentration of at least 40 mg/mL. Added 0.5 mL to the nuclear magnetic tube after transferring the dissolved solution there, acetone as internal standard. The target in this research was quantitatively analyzed using a 500 MHz nuclear magnetic resonance spectrometer from Broker (Germany), with 25°C scanning temperature.

### 2.7 Animals and experimental design

Regarding the use and treatment of animals, all relevant institutional guidelines were adhered to. For this study, wild-type male C57BL/6 mice (8 weeks, 22–24 g) were purchased from Guangzhou research into biological technology Co., LTD (China). All studies were approved by Laboratory Animals of Guangdong Pharmaceutical University (approval no. gdpulacspf2022162). All animal experiments were complied with the ARRIVE guidelines and carried out in accordance with Guidance on the operation of the Animals (Scientific Procedures) Act 1986 and associated guidelines. Mice were kept in a specialized environment that was free of pathogens, with a light/dark cycle of 12 h, a temperature of 22°C ± 1°C, and a relative humidity of 55% ± 5%, and free access to food and water. The mice were acclimatized for 1 week before induction of mucositis and the mice wellbeing was monitored at least daily throughout the experiments. The mice were randomly divided into the following three groups (n = 6 in each group): control group, 5-FU group, and 5-FU + BO group. To cause intestinal mucosal inflammation, mice in the 5-FU and 5-FU + BO groups received intraperitoneal (i.p.) injections of 40 mg/kg body weight of 5-FU every day for 5 days. The mice in the 5-FU + BO group received BO treatment (200 mg/kg body weight; intragastric (i.g.), once daily) for 9 days. The control and 5-FU groups received the same volume of vehicle for consecutive 9 days. Diarrhoea and body weight were measured every day. On day 10, the animals were killed. The length of the intestine was measured and histopathological analyses were performed on all animals.

### 2.8 Intestinal mucus stains and histopathological examination

The samples of the colon, ileum, duodenum, and stomach encased in paraffin were sliced into 4 μm thick pieces. Periodic acid-Schiff (PAS) or hematoxylin and eosin (H&E) were used to stain the sections. Under a light microscope, the histological damage of the colon was examined in the H&E-stained sections, and the histopathological scores were assessed using the scoring methodology outlined in Supplementary Table S1. Using a light microscope, the production of intestinal mucus was noted and rated in the PAS-stained sections.

### 2.9 Immunohistochemical analysis

After deparaffinizing the paraffin slices with water, they were blocked with 3% bovine serum albumin and cleaned with phosphate-buffered saline (PBS, pH 7.4). Following overnight incubation with mucin-2 antibody (1:500), the paraffin sections were treated with secondary anti-rabbit antibody (1:200) that was labeled with horseradish peroxidase. Fresh 3,3′-diaminobenzidine (DAB) was used to stain the slides, and haematoxylin was added as a post-stain. The sections were examined using a light microscope and captured on camera.

### 2.10 Immunofluorescence staining

Prior to immunofluorescence staining, the paraffin-fixed colon sections were deparaffinized, rehydrated, and washed with PBS. Following the steps of heating the antigen in a microwave, treating it with 3% H_2_O_2_, and blocking it with 3% bovine serum albumin (BSA), the sections were left overnight at 4°C to be treated with the primary antibody. Following a 50-minute incubation period at room temperature with secondary antibodies labeled with HRP, the sections were subjected to diaminobenzidine (DAB) staining and hematoxylin counterstaining.

### 2.11 Total RNA extraction and quantitative PCR

All of the reverse transcription kits were supplied by AG Biotechnology, and the reaction schedule was as follows: 30 s at 95°C, 40 cycles at 95°C for 5 s, and 30 s at 60°C. The primer sequences of target IL-1β, IL-6, IL-10 and TNF-α were shown in Supplementary Table S2. Using the 2−ΔΔCycle threshold approach, the relative abundance was computed by comparing the cycle threshold values. The relative quantification of target genes were carried out using β-Actin as an internal control.

### 2.12 Western blot

Total protein extracts were prepared with lysate buffer and uniformed by the Pierce BCA protein assay kit. The BCA Protein Assay Kit was used to quantify the isolated proteins. After being separated for electrophoresis on 10% sodium dodecyl sulphate-polyacrylamide gels (SDS-PAGE), protein samples were transferred to PVDF membranes. The protein-loaded PVDF membranes were blocked for an hour in 5% skim milk or bovine serum albumin, per the manufacturer’s instructions, and then primary and secondary antibodies were incubated on them. The Western ECL substrate was used to identify protein expression, and ImageJ was used to compare the results. The following working dilutions were made: β-actin 1:1000, p-NF-κBp65 1:1000, TLR4 1:1000 and MyD88 1:1000. Utilizing a β-actin antibody, the samples’ uniform loading was verified.

### 2.13 Analysis of the 16S RNA gene sequence

Shanghai Meiji Biomedical Technology Co., LTD (Shanghai, China) supplied the sequencing service. After total DNA was isolated from intestinal feces, the V4 region of the 16S ribosomal DNA gene was amplified by PCR using the forward primer 338F (ACT​CCT​ACG​GGA​GGC​AGC​AG) and the reverse primer 806R (GGACTACHVGGGTWTCTAAT).

### 2.14 Bioinformatic analysis

The samples were analyzed for diversity using alpha and beta diversity. The Chao, Ace, Shannon, and Simpson indices demonstrated alpha diversity, whereas principal coordinate analysis (PCoA) revealed beta diversity. Certain microbial taxa within the categories were identified by applying taxon-based analysis and linear discriminant analysis with effect size (LEfSe) with the default parameters.

### 2.15 Statistical analysis

Prism software was used for the statistical analysis, and the data was presented as mean ± SEM. With the exception of special instructions, one-way analysis of variance (ANOVA) was used to compare various groups, and the LSD test was selected for the homogeneity of variance assessment. Dunnett’s test was applied otherwise.

## 3 Results

### 3.1 Preparation and structural characterisation of BO

**FIGURE 1:**
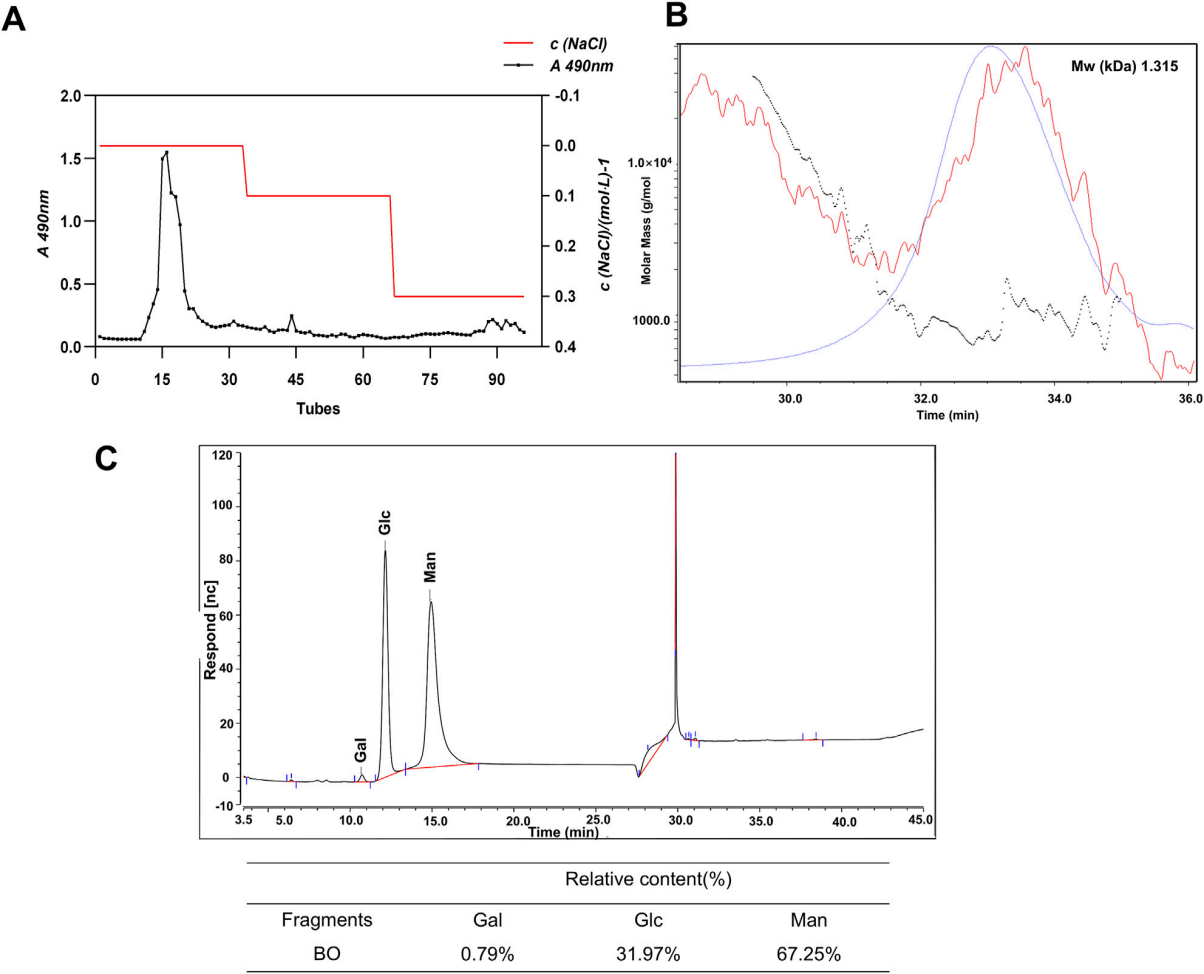
Characterization of BO. **(A)** Elution curve of BO on DEAE-52 cellulose gel column; **(B)** HPGPC spectrum; **(C)** Determination of monosacch aride composition by PMP-HPLC analysis (30 minutes is the solvent peak).

As already mentioned, BO were obtained by water extraction at 37°C, ethanol precipitation and acid hydrolysis. A DEAE 52 cellulose ion exchange column was used to purify the raw sugar. As can be seen in Figure 1A, there was a clear elution peak during the elution of distilled water. The elution of distilled water was freeze-dried and the resulting white powder was analysed. The relative molecular weight of BO is 1.315 kDa (Figure 1B). Analysis of the monosaccharide composition showed that BO consisted mainly of a small amount of Gal, Glc and Man and the molar ratio was 1.0:40.5:85.1, indicating that BO was a neutral sugar (Figure 1C).

### 3.2 Methylation analysis of BO

BO was methylated using MeI in order to further clarify the linking patterns of the sugar residues, and the linkage patterns were then examined using GC-MS (Supplementary Figure S1). Five different linkage types were found in the BO, according to the results: t-Man(p), t-Glc(p), 4-Man(p), 4-Glc(p), and 3,4-Man(p). BO were mainly composed of five glycosidic residues: 5-di-O-acetyl-2,3,4,6-tetra-O-methyl mannitol, 5-di-O-acetyl-2,3,4,6-tetra-O-methyl glucitol, 4,5-tri-O-acetyl-2,3,6-tri-O-methyl mannitol, 4,5-tri-O-acetyl-2,3,6-tri-O-methyl glucitol and 3,4,5-tetra-O-acetyl-2,6-di-O-methyl mannitol (Table 1) (Supplementary Figure S2).

**TABLE 1:** Methylation analysis of BO.

Linkage type	Methylated sugars	Mw	Molar ratio (mol%)
t-Man	5-di-O-acetyl-2,3,4,6-tetra-O-methyl mannitol	323	16
t-Glc	5-di-O-acetyl-2,3,4,6-tetra-O-methyl glucitol	323	11
4-Man	4,5-tri-O-acetyl-2,3,6-tri-O-methyl mannitol	351	49
4-Glc	4,5-tri-O-acetyl-2,3,6-tri-O-methyl glucitol	351	21
3,4-Man	3,4,5-tetra-O-acetyl-2,6-di-O-methyl mannitol	379	3

### 3.3 NMR analysis

**FIGURE 2:**
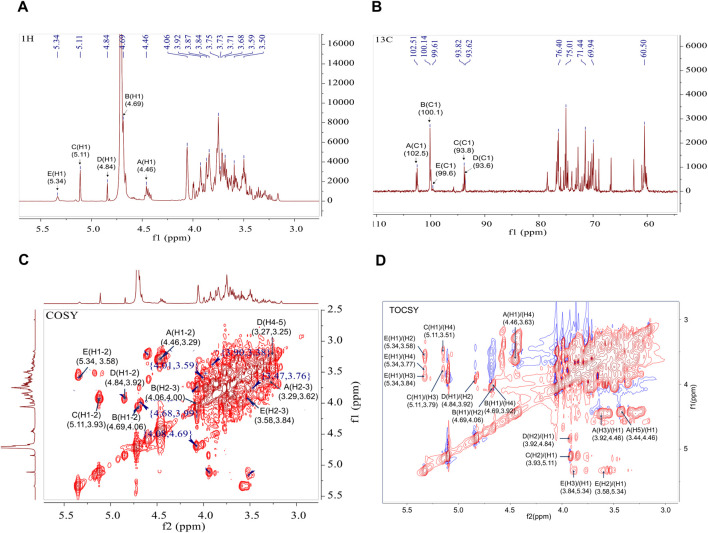
1D and 2D NMR spectra of BO. **(A)** 1H NMR spectrum; **(B)** 13C NMR spectrum; **(C)** 1H-1H COSY spectrum; **(D)** 1H-1H TOCSY spectrum.

The structural characteristics of BO were further examined in this work using two-dimensional NMR (COSY, HSQC, HMBC, NOESY, and TOCSY) and one-dimensional NMR (1H and 13C). The configurations of α and β in BO are displayed by the 1H and 13C signals, which are in the range of δ H4.3∼5.4 and δ C93 ∼103 ppm, respectively (Figures 2A, B). Five distinct peaks can be recognized based on the cross-peak signal in the HSQC (Supplementary Figure S3A), 1H NMR, and 13C NMR spectrum: δ 4.46/102.5, 4.69/100.1, 5.11/93.8, 4.84/93.6, and 5.34/99.6 ppm, compared with published reports. The isomeric signal of the β-configuration in BO can be identified by the chemical shifts of δ 4.46/102.5 and δ 4.69/100.1 ppm (Zhang, et al., 2021; Zhu et al., 2023), whereas the signal of the α-configuration in BO can be identified by the chemical shifts of δ 5.11/93.8, 4.84/93.6, and 5.34/99.6 ppm (Li et al., 2023; Rong et al., 2021; Zeng et al., 2020). A cross signal in the COSY spectrum (Figure 2C) is utilized to determine the H1/H2 of each residue. The H1/H2 of the A-E residues are represented by the cross peaks of δ 4.46/3.29, 4.69/4.06, 5.11/3.93, 4.84/3.92, and 5.34/3.58 ppm, successively. The COSY and TOCSY spectrum (Figure 2D) display signals δ 4.46/3.29, 3.29/3.62, 3.62/3.63, 3.63/3.44 and 3.44/3.57 (3.49) ppm that are associated with residue A’s H1-H6 group. The HSQC spectrum signals δ 72.9/3.29, 74.0/3.62, 78.5/3.63, 75.2/3.44 and 62.5/3.57 (3.49) ppm correspond to residue A’s C2/H2-C6/H6. Based on the methylation studies presented in research and literatures, residue A was determined to be →4)-β-Manp-(1→. Similarly, the COSY and TOCSY spectrum of residue B assign δ 4.69/4.06, 4.06/4.00, 4.00/3.92, 3.92/3.84 and 3.84/3.68 (3.92) ppm to H1-H6. The HSQC spectrum’s δ 4.06/70.0, 4.00/70.5, 3.92/76.8, and 3.84/71.1 ppm formants are attributed to residue B’s C2/H2-C6/H6, indicating that residue B is →4)-β-Glcp-(1→. Assigning signals to the glucoside bond C-E follows the same procedure; NMR data published in the literature is used to assign the 1H and 13C signals indicated in Table 2.

**TABLE 2:** Chemical shift of each sugar residue 1H and 13C.

Code	Glycosyl residues	Chemical shifts (ppm)					
		H1/C1	H2/C2	H3/C3	H4/C4	H5/C5	H6/C6
A	→4)-β-Man*p*-(1→	4.46	3.29	3.62	3.63	3.44	3.57, 3.49
		102.5	72.9	74.0	78.5	75.2	62.5
B	→4)-β-Glc*p*-(1→	4.69	4.06	4.00	3.92	3.84	3.68, 3.92
		100.1	70.0	70.5	76.8	71.1	60.2
C	α-Man*p*-(1→	5.11	3.93	3.79	3.51	3.73	3.49, 3.60
		93.8	70.3	71.0	72.0	71.6	62.5
D	α-Glc*p*-(1→	4.84	3.92	3.50	3.27	3.25	3.71, 3.65
		93.6	73.3	72.3	73.4	73.0	60.5
E	→3,4)-α-Man*p*-(1→	5.34	3.58	3.84	3.77	3.66	3.77, 3.95
		99.6	73.1	76.8	76.6	72.4	60.4

HMBC and NOESY spectrum helped to further clarify the binding mechanism of the sugar residues in BO (Supplementary Figures S3B, C). Correlation peaks in the HMBC spectrum are located at δ 4.46/78.49 (A-H1/A-C4), 4.46/76.79 (A-H1/B-C4), 4.69/76.79(B-H1/B-C4), 4.69/76.78 (B-H1/E-C3) and 3.84/100.14 (E-H3/B-C1) ppm, signifying that →4)-β-Manp-(1→4)-β-Manp-(1→, →4)-β-Manp-(1→4)-β-Glcp-(1→, →4)-β-Glcp-(1→4)-β-Glcp-(1→ and →4)-β-Glcp-(1→3,4)-α-Manp-(1→. The linking sequence of each individual residue in the polysaccharide could be determined as follows using the NOESY spectrum: There was a cross peak δ5.11/3.63 ppm between the sugar residues C-H1 and A-H4, and a cross peak δ4.84/3.77 ppm between D-H1 and E-H4. Therefore, from the one-dimensional and two-dimensional NMR information and methylation analysis, it can be concluded that the polysaccharide is mainly linked by →4)-β-Manp-(1→, →4)-β-Glcp-(1→ and a small amount of →3,4)-α-Manp-(1→ to form the main chain. The branched chain is mainly composed of α-Glcp-(1→ connected at the O-4 position of the sugar residue →3,4)-α-Manp-(1→.

### 3.4 BO alleviated the symptoms and histopathological damage of 5-FU-induced intestinal mucositis in mice

**FIGURE 3:**
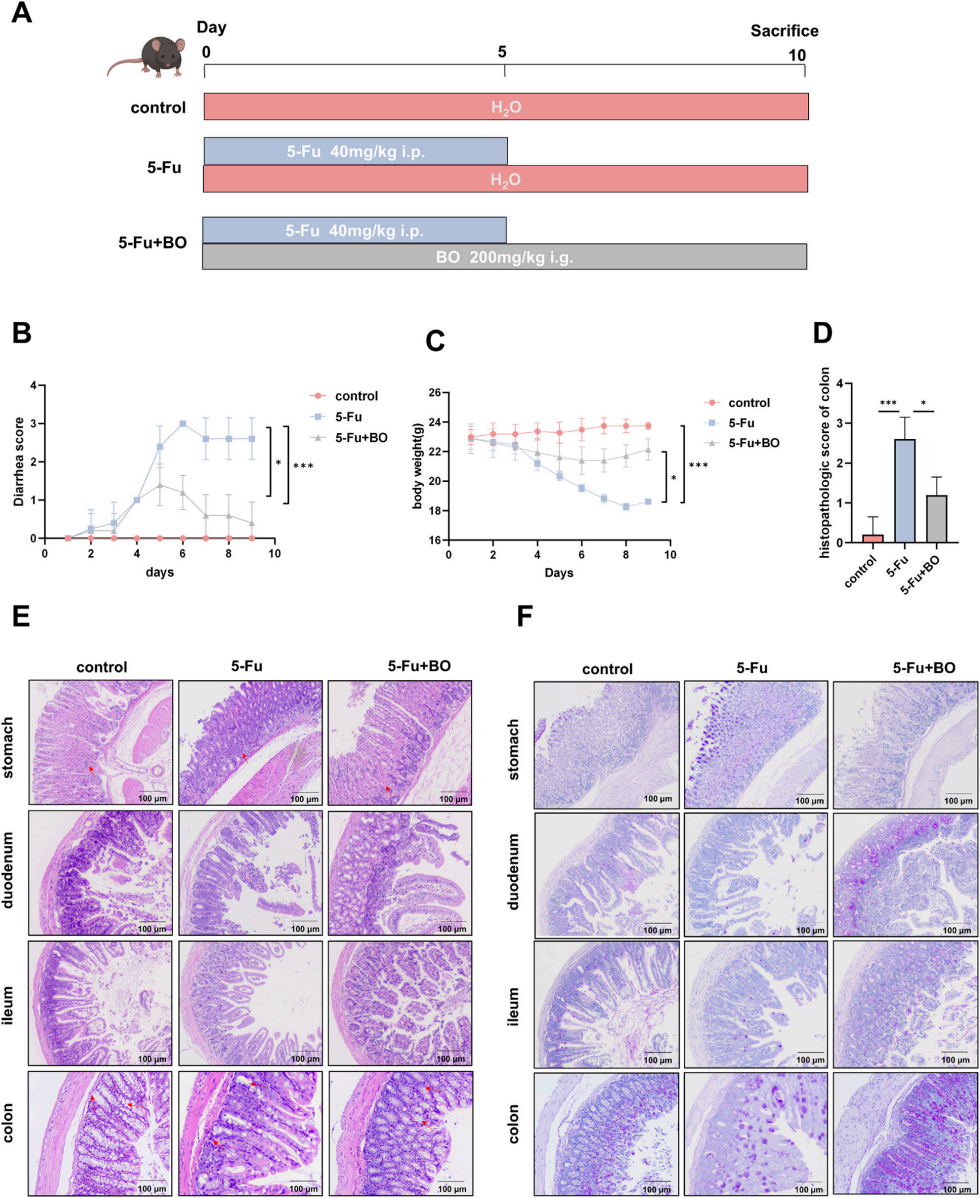
Effects of BO on symptoms and histopathological damage in mice with CIM (n = 6). **(A)** The establishment of the CIM model in mice and the administration of BO; **(B)** Diarrhoea score; **(C)** Body weight; **(D)** Histopathological scores were evaluated by the results of H&E staining; **(E)** H&E staining of colon, ileum, duodenum and stomach; **(F)** PAS staining of colon, ileum, duodenum and stomach; *p < 0.05,***p < 0.001.

In this study, to determine the protective effect of BO against CIM, we induced intestinal injury by intraperitoneal injection in mice of 5-FU (40 mg/kg) for 5 days (Figure 3A). The mice in the 5-FU group had severe diarrhea and a marked decrease in weight as compared to the normal group. However, BO were able to greatly attenuate the diarrhea and weight loss driven by 5-FU (Figures 3B, C). Interestingly, we compared the damage to the stomach, duodenum, ileum and colon and found that 5-FU damaged the stomach and colon most significantly. We next selected the colon for further investigations. HE staining was used to evaluate the histopathological changes. According to the results, a semi-quantitative analysis of the pathological lesions of the colon was performed. The findings demonstrated that the colon of the 5-FU group had clear tissue damage and inflammatory reactions, including goblet cells, crypt destruction, and neutrophil and monocyte infiltration. In contrast, the normal group had no histological abnormalities. However, treatment with BO improved the above-mentioned colon damage and inflammatory cell infiltration (Figures 3D, E). As we known, the goblet cells’ produced mucus is crucial in defending the colon against harmful bacteria. The results of PAS staining of the 5-FU group showed that, compared to the normal group, the secretion of mucus and goblet cells were significantly reduced in the 5-FU group and BO intervention was able to significantly attenuate the 5-FU-induced decrease in mucus and goblet cells (Figure 3F). These results suggested that BO can significantly improve 5-FU-induced colonic mucosal inflammation and alleviate colonic damage.

### 3.5 BO preserved the integrity of the intestinal barrier and decreased intestinal inflammation through the TLR4/MyD88/NF-κB pathway

**FIGURE 4:**
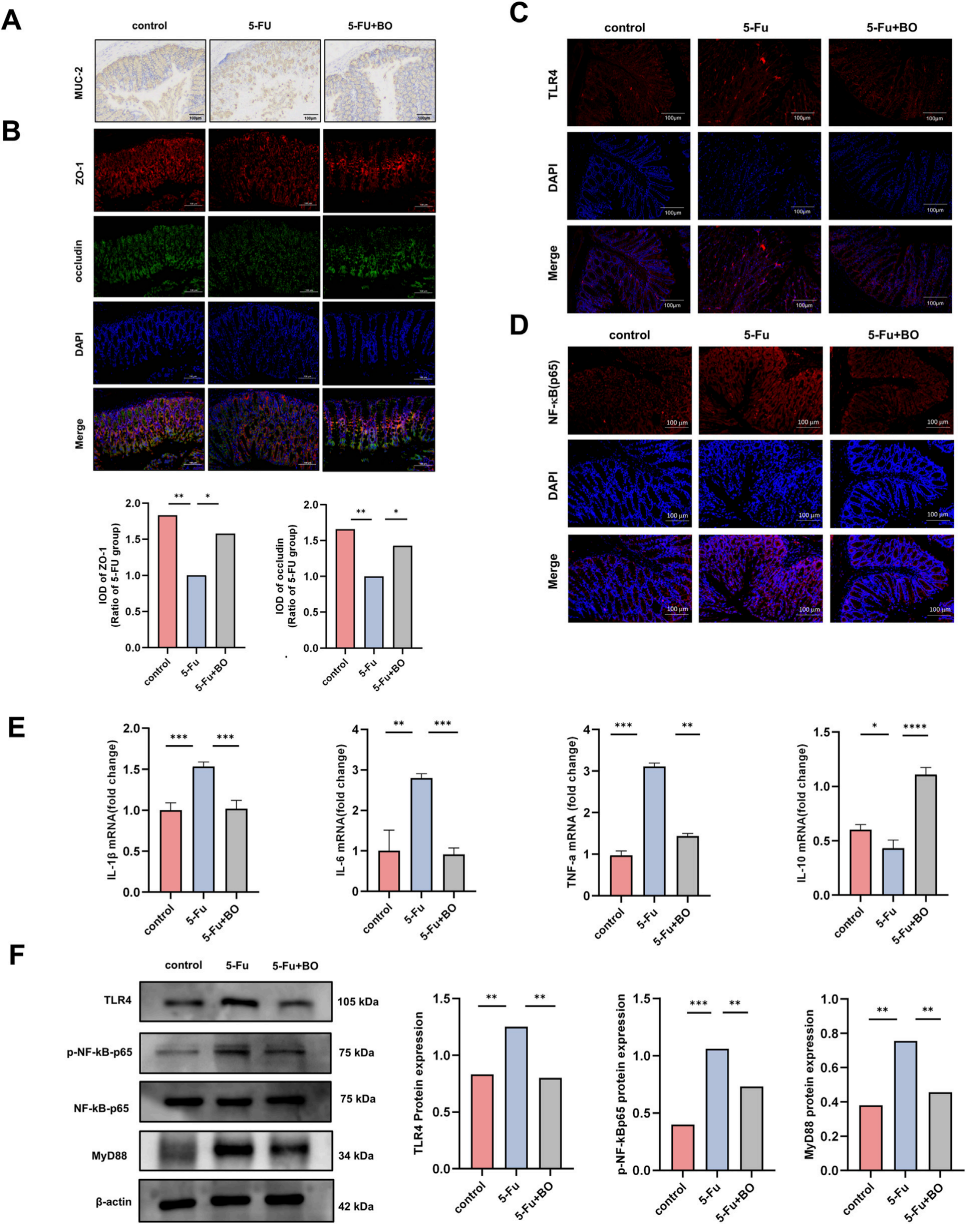
Effect of BO on intestinal barrier and inflammation. **(A)** Immunohistochemistry for MUC-2 in colon; **(B)** The immunofluorescence of ZO-1 and occludin; **(C, D)** Expression of TLR4 and NF-κB (p65) protein (200 ×). Values were represented by the mean ± SEM; **(E)** Levels of IL-1β, IL-6, TNF-α, IL-10; **(F)** Expressions of TLR4, ρ-NF-κB-p65, NF-κB-p65 and MyD88 proteins. ***P < 0.001, **P < 0.01, *P < 0.05.

The balance of mucus production in the intestine is the basis for the maintenance of normal organ function. We detected the content of mucin-like glycoprotein in the colon by PAS staining (Figure 3F). In addition, the expression of MUC-2 was detected by immunohistochemistry (Figure 4A). Results displayed that BO improved the production of intestinal mucus. The intestinal mucosal barrier and the passage of pathogens and poisons through epithelial cells are both regulated by tight junction proteins. Investigating the tight junction protein ZO-1 and occludin expression (Figure 4B), we discovered that 5-FU treatment severely reduced the expression of these proteins, while BO dramatically reversed this impact. To investigate the mechanism of protective effects of CIM, we measured the cytokine levels in the colons, assessing both mRNA and protein expressions. TLR4 expression was upregulated by 5-FU treatment, according to immunofluorescence data (Figure 4C). And NF-κB was found downstream of the TLR4 signaling pathway. TLR4 can also attract a downstream signaling protein called myeloid differentiation factor 88 (MyD88), which allows it to transfer NF-κB to the nucleus and trigger the production of inflammatory mediators like IL-1β and TNF-α. The findings demonstrated that 5-FU greatly increased NF-κB in the nucleus (Figure 4D). The expressions of TNF-α, IL-1β, IL-6 and IL-10 were measured in order to examine the impact of BO on inflammatory factors. The findings demonstrated that the 5-FU group had considerably higher levels of pro-inflammatory cytokines, such as IL-1β, IL-6, and TNF-α. Nonetheless, BO reduced the expression levels of these inflammatory factors. The anti-inflammatory factor IL-10 levels in the control and model groups did not differ substantially, while IL-10 levels following BO therapy increased significantly (P < 0.001) (Figure 4E). As a member of the NF-κB family, the expression of the phosphorylated protein p-NF-κB-p65 can be used to determine whether the NF-κB signaling pathway has been activated. Western blot examination revealed that the aberrant development of p-NF-κB-p65 and MyD88 proteins by 5-FU was considerably suppressed by BO treatment (P < 0.001 compared to 5-FU) (Figure 4F).

### 3.6 Effect of BO on the analysis of microbial diversity

In this study, total DNA was extracted from feacal samples of the control, 5-FU and BO groups and 16S rRNA sequencing was performed to analyse the gut flora. A total of 5,1147,8101 high quality reads with an average sequence length of 420 (n = 6) were collected. Biostatistical analysis was performed for the OTUs whose similarity level was 97% (Supplemetary Table S3). Pan OTU is the sum of OTU contained in all samples, and core OTU refers to the number of OTU shared by all samples. The pan/core species curve became flat as the number of species grew, suggesting that the sequencing sample size was adequate (Supplementary Figures S4A, B).

**FIGURE 5:**
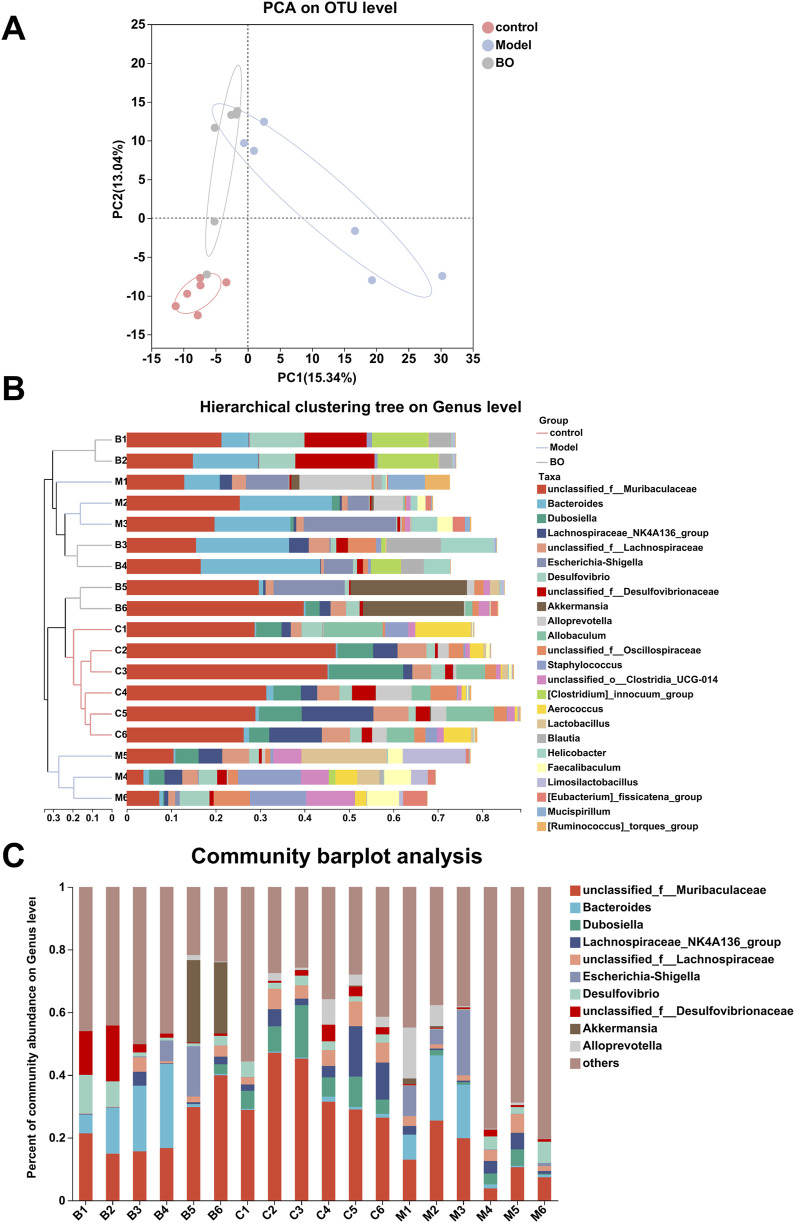
Species analysis. **(A)** PCA analysis; **(B)** Hierarchical clustering tree on Genus level; **(C)** Community barplot analysis at Genus level.

The analysis of alpha diversity reflected the diversity of species within the group. The richness of the microbial community was analysed by determining the Sobs and Chao index, the diversity of the community was described by determining the Shannon and Simpson index. In contrast to the control group, the Chao, Sobs, and Shannon indices declined in the BO group, whereas the Simpson index improved (Suppleemtary Figures S4C–F). Comparisons between the beta species diversity of various microbial communities were made in order to explore the similarities and variations in the community makeup of various microbial communities. The varying sample branch length clustering within the groups reflected the varying distribution of species richness both within and between the categories. There was a difference in the community makeup between the control group and the model group. The genus-level cluster analysis results demonstrated that the flora compositions of the BO group and the control group were similar and notably different from those of the model group, with no significant separation found between them. It was evident that BO may successfully manage the balance of intestinal flora and influence the intestinal flora problem caused by 5-FU (Figures 5A, B).

**FIGURE 6:**
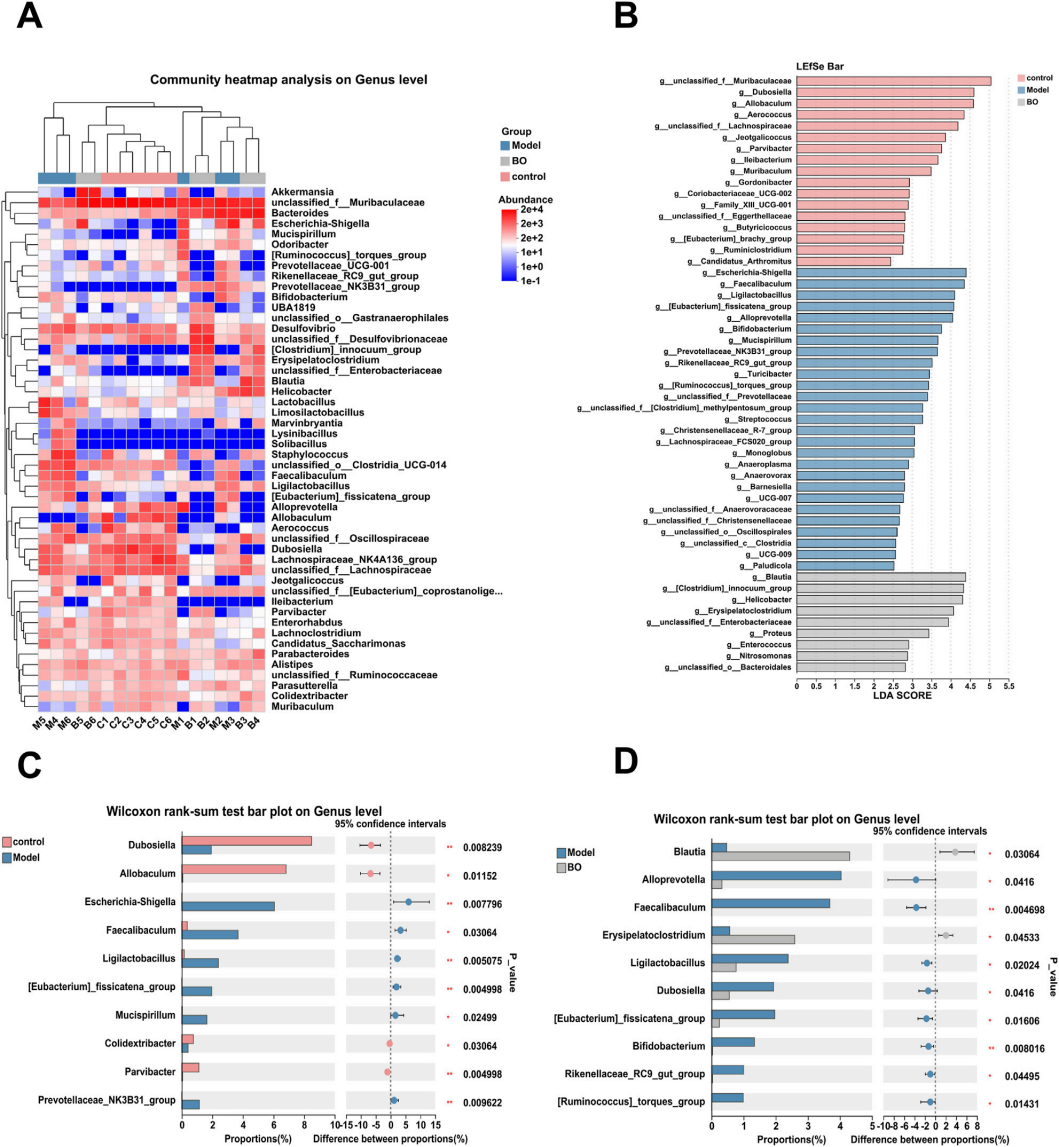
Relative Abundance Analysis of Sample. **(A)** Clustering heat map analysis on Genus level; **(B)** Difference test among multiple groups; **(C)** Difference test between Control group and Model group; **(D)** Difference test between Model group and BO group.

Visualisation methods such as bar charts and heat maps were used to comprehensively analyse the species diversity of the samples at different classification levels. In the three experimental groups, Firmicutes and Bacteroidetes dominated the Bacteroidetes phylum. With a higher relative abundance of Bacteroidetes and a lower relative abundance of Firmicutes, the model group displayed more differences than the control and BO groups. The bacterial imbalance caused by 5-FU showed obvious individual differences, and the administration of BO could alleviate this difference at the phylum level and increase the relative abundance of Firmicutes. These findings suggested that by adjusting the quantity of Firmicutes and Bacteroidetes, BO can modify the gut microbial structure of mice with intestinal mucosal inflammation at the phylum level (Supplementary Figure S5A). At the family level, the control group had the highest relative abundance of Muribaculaceae, followed by Erysipelotrichaceae and Lachnospiraceae. The relative abundance of Muribaculaceae in the model group was lower than in the normal group (Supplementary Figure S5B). At the level of the genus unclassified_f__Muribaculaceae, a branch of Muribaculaceae, the administration of BO was able to reverse the effects of 5-FU on the microflora in mice and increase the relative abundance of unclassified_f__Muribaculaceae. Additionally, the experimental group treated with 5-FU had a considerable decrease in the abundance of Dubosiella, which was the second dominating bacteria in the control group (Figures 5C, 6A). In summary, BO increased the population of beneficial bacteria and altered the ecology at the three levels of phylum, family, and genus.

After clarifying the regulatory effect of BO on the gut microbiota, a further analysis of the differences in genus levels between all groups was performed (Figure 6B). Compared with the normal group, the abundance of unclassified_f__Muribaculaceae, Dubosiella, unclassified_f__Lachnospiraceae and Allobaculum decreased in the model group. The relative abundance of Escherichia-Shigella, Faecalibaculum, Ligilactobacillus and other bacteria increased (Figure 6C). The relative abundances of Blautia and Erysipelatoclostridium in the BO group were substantially higher than those of the model group, although the abundances of the species Alloprevotella and Faecalibaculum were significantly lower (Figure 6D).

## 4 Discussion

The structure of the neutral sugar obtained by DEAE-52 cellulose purification was analysed. The monosaccharides of BO were Man, Glc and Gal, and the molecular weight was 1.315 kDa. The sugar chain consisted mainly of t-Man(p), t-Glc(p), 4-Man(p), 4-Glc(p) and 3, 4-Man (p).

In this study, it was found that the weight, diarrhoea, shortened colon and other symptoms of 5-FU-induced intestinal mucosal inflammation in mice improved after BO treatment, suggesting that BO have a potential therapeutic effect on 5-FU-induced intestinal mucositis in mice. Gastrointestinal illnesses can interfere with intestinal flora balance, which is a crucial component of gastrointestinal health. In gastrointestinal disorders such IBD (Lee and Chang, 2021), radiation-induced mucositis (Al-Qadami, et al., 2019; Oh, et al., 2021) and chemotherapy-induced mucositis, the intestinal microbiota is crucial (van Vliet, et al., 2010). Research has demonstrated that anaerobic bacterial population and microbial diversity are both decreased during chemotherapy. It is reported that 5-FU has been shown to reduce the formation of *Fusobacterium* nucleatum, which may associate cancer patients’ prognosis (LaCourse, et al., 2022). 5-FU also raised the number of Verrucomicrobia and Actinobacteria in the contents of the feces or cecum while decreasing the proportion of Proteobacteria, Tenericutes, Cyanobacteria, and Candidate division TM7(Li, et al. 2017). After treatment with 5-FU, the microbial community in mice changed, and the number of harmful bacteria such as Escherichia-Shigella *shigella* and Mucispirillum in mice increased. It is known that Escherichia-Shigella *shigella* can colonise the epithelium of the small intestine through adhesins and then release enterotoxin into the host’s epithelial cells, causing diarrhoea (Zhang, et al., 2022b). In this study, the BO group had a considerably higher relative incidence of Blautia when compared to the model group. According to earlier research, patients with inflammatory bowel illness had a marked decrease in the quantity of Blautia in their cecal mucosa microbiota. Blautia is a bacterial genus that functions as an obligatory anaerobic symbiosis that increases the activity of gut regulatory T cells and generates SCFA. BO play an important role in maintaining the ecological balance in the gut and preventing inflammation (Liu X. et al., 2021), suggesting that BO may regulate the inflammatory process in the gut by influencing the relative abundance of inflammation-relevant bacteria.

A “five-step model” of the pathophysiology of mucositis was proposed by Sonis et al. in earlier research. The steps include initiation, signal amplification, ulcer with inflammation, and healing stages (Sonis, 2004). Clinical studies have confirmed that CIM can cause intestinal flora disruption, which in turn can exacerbate mucositis through interaction with TLRs (Wei, et al., 2021). TLR4, the main receptor recognised by LPS, recruits adaptor proteins via the MyD88-dependent and MyD88-independent pathways to activate NF-κB and enhance its effect (Lupi, et al., 2020). NF-κB has been associated with many autoimmune diseases, cancer, viral replication, apoptosis, and inflammation. It is an essential organiser of the immune system’s innate and adaptive defences. In this study, the inhibitory effect of BO on intestinal mucosal inflammation in mice was largely dependent on the TLR4/MyD88/NF-κB signalling pathway. NF-κB may initiate an inflammatory response and cause a release of pro-inflammatory mediators, which in turn can activate and differentiate immune cells (Yu, et al., 2020). In this experiment, inflammatory cytokines TNF-α, IL-1β, and IL-6 can be decreased by BO. The key proinflammatory cytokines, such as IL-1β, IL-6, and TNF-α, are extensively involved in the inflammatory process. Among these, IL-1β and TNF-α are implicated in the activation of the standard NF-κB signaling pathway (Barnabei, et al., 2021). IL-6 has a wide range of biological functions, especially in the development of inflammatory illnesses (Guo, et al., 2021). The cytokine IL-10 is an anti-inflammatory mediator that can protect the host from overreaction to pathogens and microorganisms (Saraiva, et al., 2020) and is essential for the maintenance of healthy epithelial homeostasis (Nguyen, et al., 2021). IL-10 can also limit IKK activity, which in turn prevents IκBα from being phosphorylated and degraded. As a result, NF-κB is unable to enter the nucleus and perform its role (Driessler, et al., 2004; Schottelius, et al., 1999). It has been reported that mice lacking IL-10 experience inflammatory bowel illness (Zegarra Ruiz, et al., 2022). According to this study, BO can increase IL-10 expression. It improves the inflammation of the intestinal mucosa in a beneficial way.

In the current study, increased intestinal permeability and widespread inflammation were appeared in 5-FU-induced mice (Deng, et al., 2021; Xia, et al., 2022). Multiprotein complexes like claudin, occludin, ZO-1, and other cytoplasmic and transmembrane proteins make up the tight junction (TJ). Various external cues dynamically govern changes in paracellular permeability and TJ barrier function. Proinflammatory cytokines can penetrate deeper into the body once the TJ barrier breaks down, perhaps leading to the activation of the mucosal immune system. Tissue injury and ongoing inflammation result from this (Suzuki, 2013). According to our findings, the mice in the model group had greater intestinal permeability and less expression of occludin and ZO-1. BO upregulated the expression of ZO-1 and occludin, further stabilizing the intestinal epithelial barrier. The mucus layer is a component of the non-immune part of the intestinal barrier, a complex structure composed of mucous proteins that form viscoelastic gels, protecting the gastrointestinal epithelium from damage (Bergstrom, et al., 2016). MUC-2 is a secreted protein produced by epithelial goblet cells and is later described as a key component of mucus (Liu et al., 2020). Previous studies have shown that the reduction of MUC-2 levels may be associated with various intestinal diseases (Kang, et al., 2022). We were able to ascertain that the goblet cells in the model group had very little MUC-2 production or secretion by using PAS and IHC studies. This implies that 5-FU seriously harmed the goblet cells, either directly or indirectly. Goblet cells’ biological function progressively returned following BO treatment, and there was an increase in MUC-2 production, which helped preserve the epithelial barrier.

## 5 Conclusion

To summarise, according to the results of HPGPC, methylation and NMR analysis, BO is an oligosaccharide with a molecular weight of 1.315 kDa consisting mainly of Gal, Glc and Man. Our results had shown that BO effectively combated intestinal mucosal inflammation in mice. The potential mechanisms were attenuation of the inflammatory response, protection of the intestinal mucosal barrier and improvement of the intestinal microbial community. This anti-inflammatory process was mainly mediated by the TLR4-NF-κB/Myd88 signalling pathway, so BO can be used as a new dietary supplement for the treatment of CIM.

## Data Availability

The datasets presented in this study can be found in online repositories. The names of the repository/repositories and accession number(s) can be found in the article/Supplementary Material.
