# Supplementary figures and images for: Bletilla oligosaccharides improved 5-fluorouracil-induced intestinal mucositis in mice by activating NF-κB signalling pathway and regulating intestinal microbiota
Source: Front Pharmacol. 2025 Mar 13;16:1526274. doi: 10.3389/fphar.2025.1526274 (PMC11965902; doi:10.3389/fphar.2025.1526274)

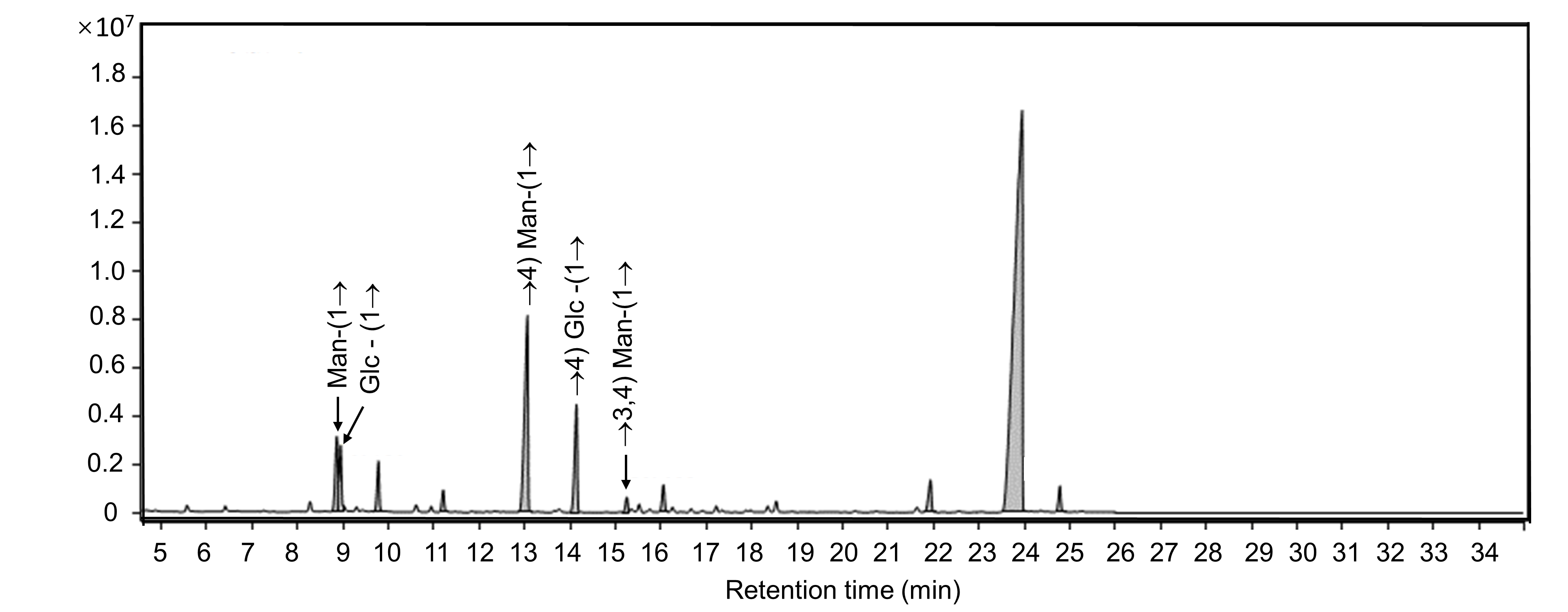

Supplement: Supplementary file 1 [file DataSheet2.zip › figureS1.tif]

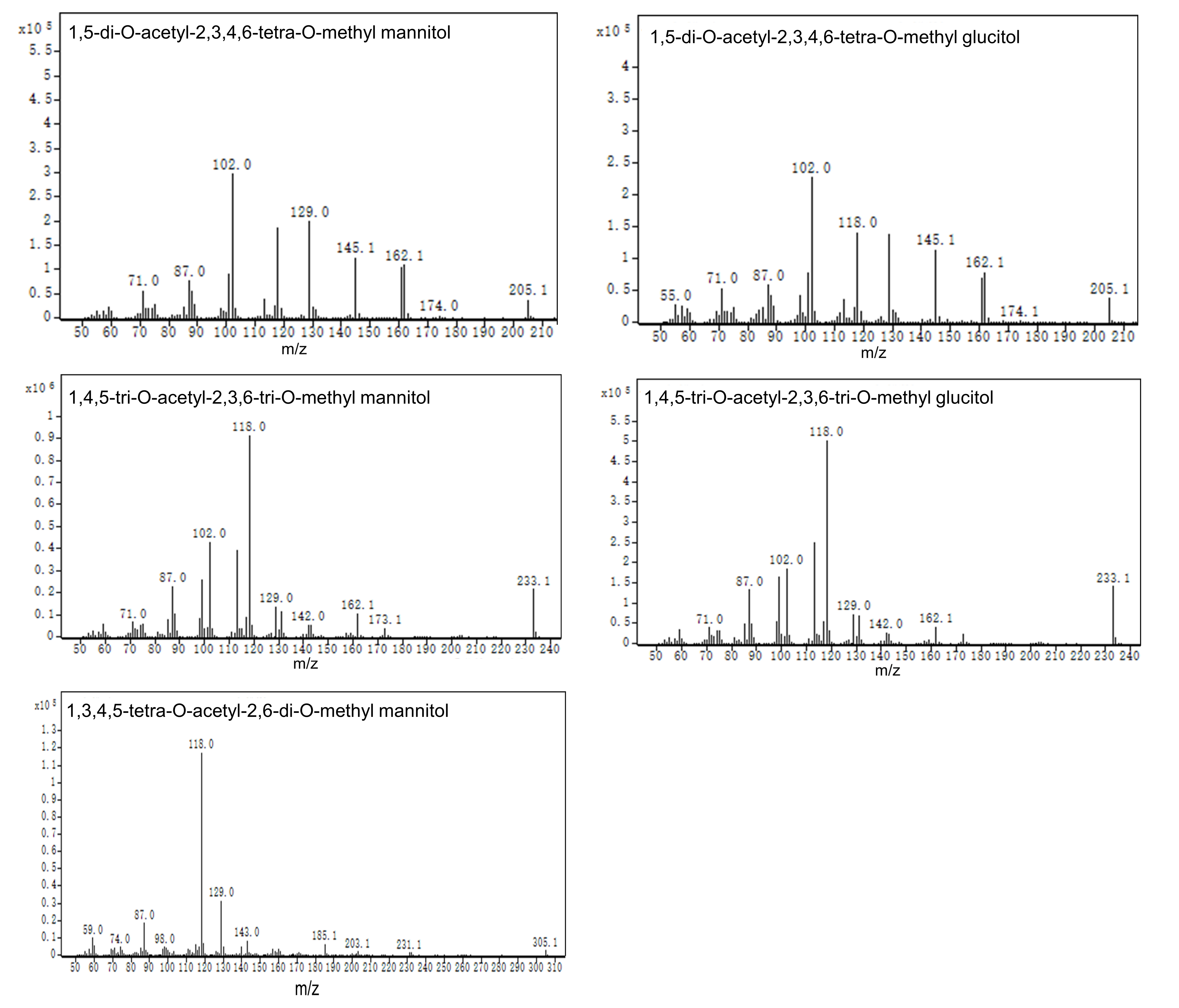

Supplement: Supplementary file 1 [file DataSheet2.zip › figureS2.tif]

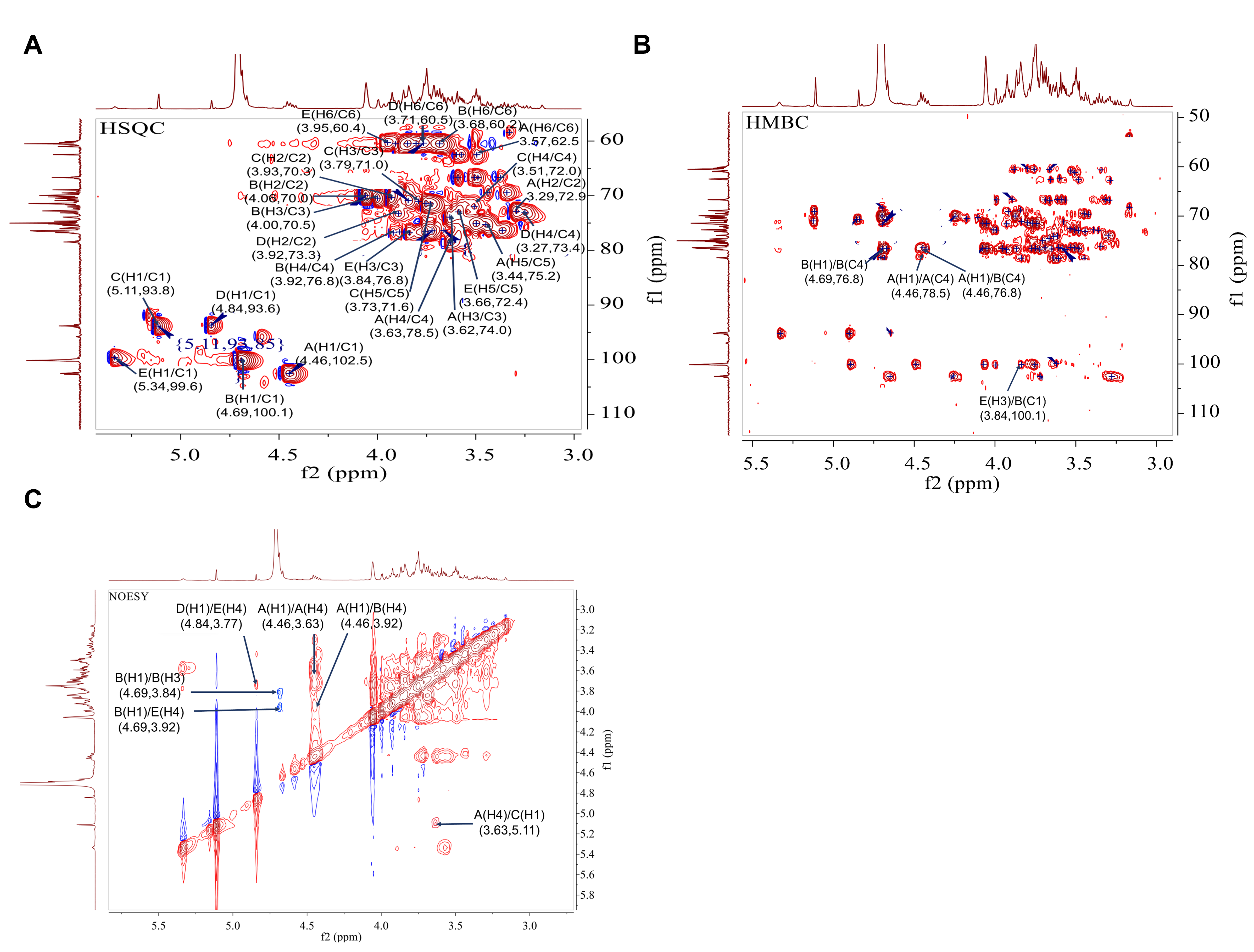

Supplement: Supplementary file 1 [file DataSheet2.zip › figureS3.tif]

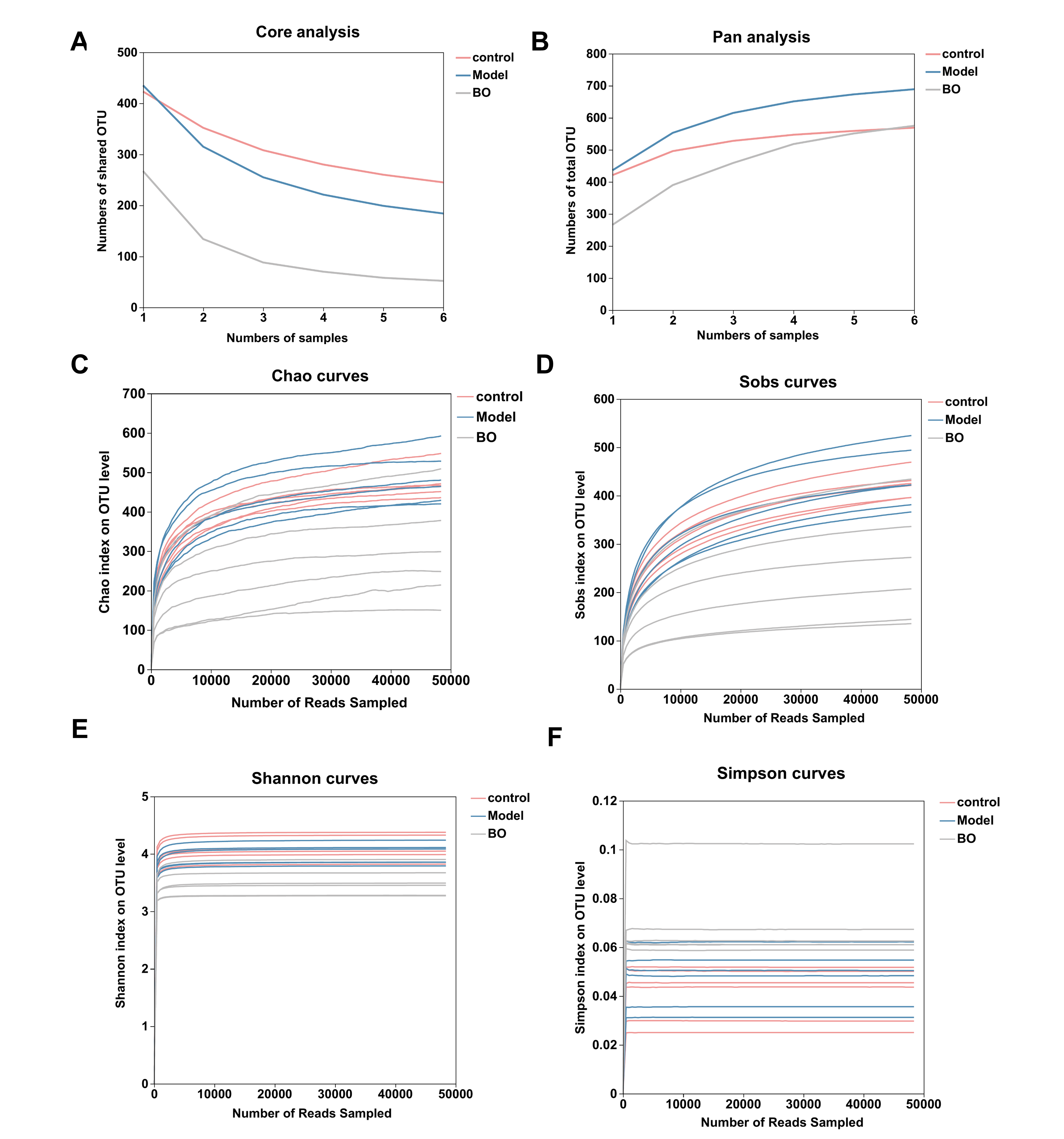

Supplement: Supplementary file 1 [file DataSheet2.zip › figureS4.tif]

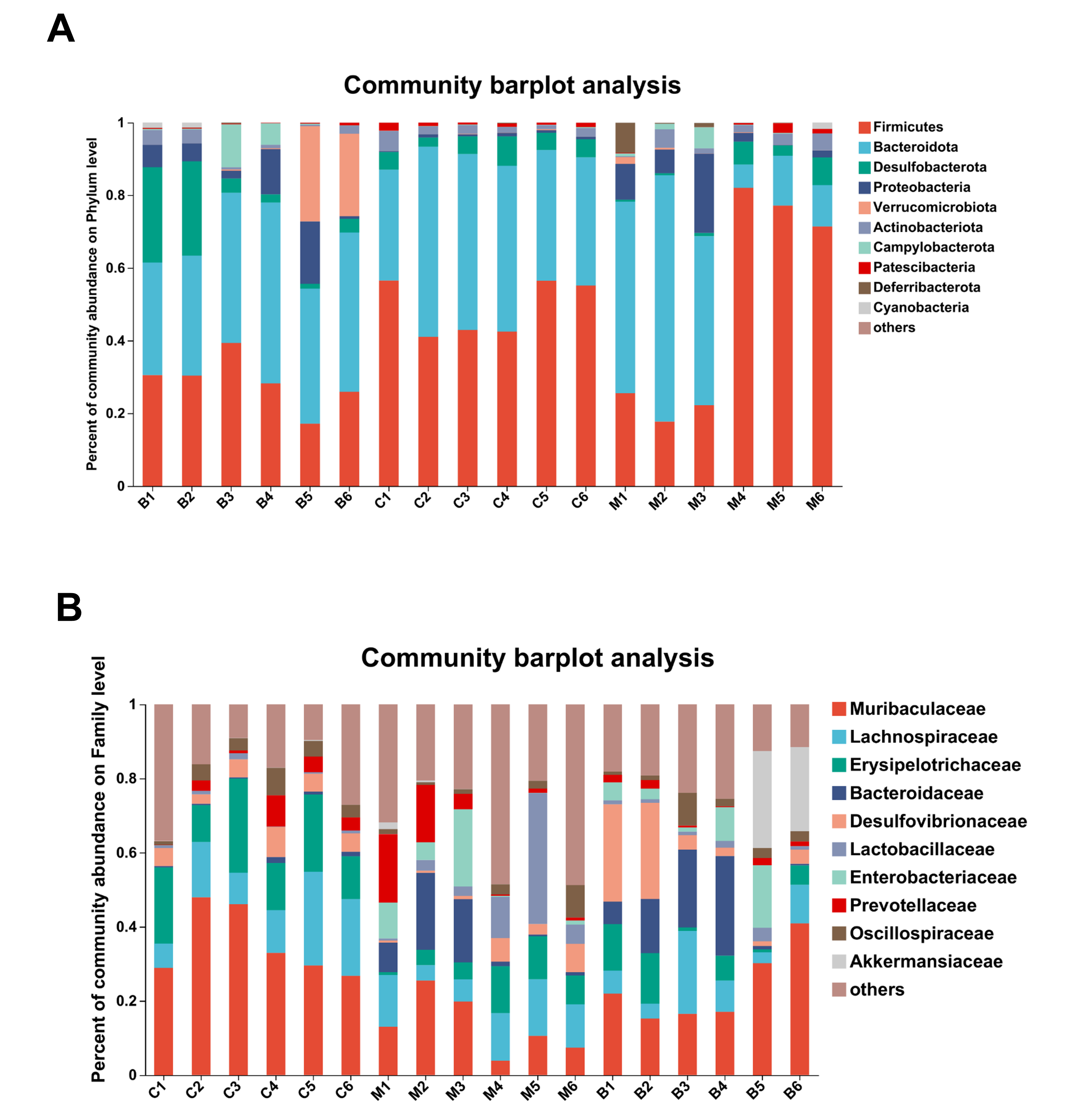

Supplement: Supplementary file 1 [file DataSheet2.zip › figureS5.tif]
