# Supplementary material for: Bletilla oligosaccharides improved 5-fluorouracil-induced intestinal mucositis in mice by activating NF-κB signalling pathway and regulating intestinal microbiota
Source: Front Pharmacol. 2025 Mar 13;16:1526274. doi: 10.3389/fphar.2025.1526274 (PMC11965902; doi:10.3389/fphar.2025.1526274)
Supplement: Supplementary file 2 [file DataSheet1.docx]

**Supplementary Materials**

**Bletilla oligosaccharides improved 5-fluorouracil-induced intestinal mucositis in mice by activating NF-κB signalling pathway and regulating intestinal microbiota**

Qiuxiong Yin^1^, Xinran Li^1^, Yanli Xiong^1^, Yupeng Jiang^1^, Shengsuo Ma^2,**^, Guoqiang Qian^1,*^

^1^ School of Traditional Chinese Medicine, Guangdong Pharmaceutical University, Guangzhou, China

^2^ Department of Experimental Research, Sun Yat-sen University Cancer Center, Guangzhou, China

^*^ Correspongding Author: Guoqiang Qian

E-mail address: tgqqian@gdpu.edu.cn

^**^ Co-correspongding Author: Shengsuo Ma

E-mail address: Mass@sysucc.org.cn

**Table S1 Colonic microstructure damage score**

| Scores | Degree of colon damage |
| --- | --- |
| 0 | normal |
| 1 | Crypt slightly deformed , with slight inflammatory infiltration |
| 2 | Crypt obviously deformed , with some inflammatory infiltration |
| 3 | Absence of crypt structure , with marked inflammatory infiltration |
| 4 | Absence of crypt structure , with severe inflammatory infiltration |

**Table S2** **q-PCR primer sequence**

| species | Gene name | Forward primer (5'->3') | Reverse primer (5'->3') |
| --- | --- | --- | --- |
| Mus | IL-10 | AAGCTCCAAGACCAAGGTGTC | ACGAGGTTTTCCAAGGAGTTGT |
| Mus | IL-6 | CTGCAAGAGACTTCCATCCAG | AGTGGTATAGACAGGTCTGTTGG |
| Mus | IL-1β | AATCTCGCAGCAGCACATCA | GGAAGGTCCACGGGAAAGAC |
| Mus | TNF-a | CGGGCAGGTCTACTTTGGAG | ACCCTGAGCCATAATCCCCT |

**Table S3**

**Basic information of fecal flora**

| Amplified Region | Samples | Sequences | Bases(bp) | Average Length |
| --- | --- | --- | --- | --- |
| 338F_806R | 18 | 1217435 | 511478101 | 420 |


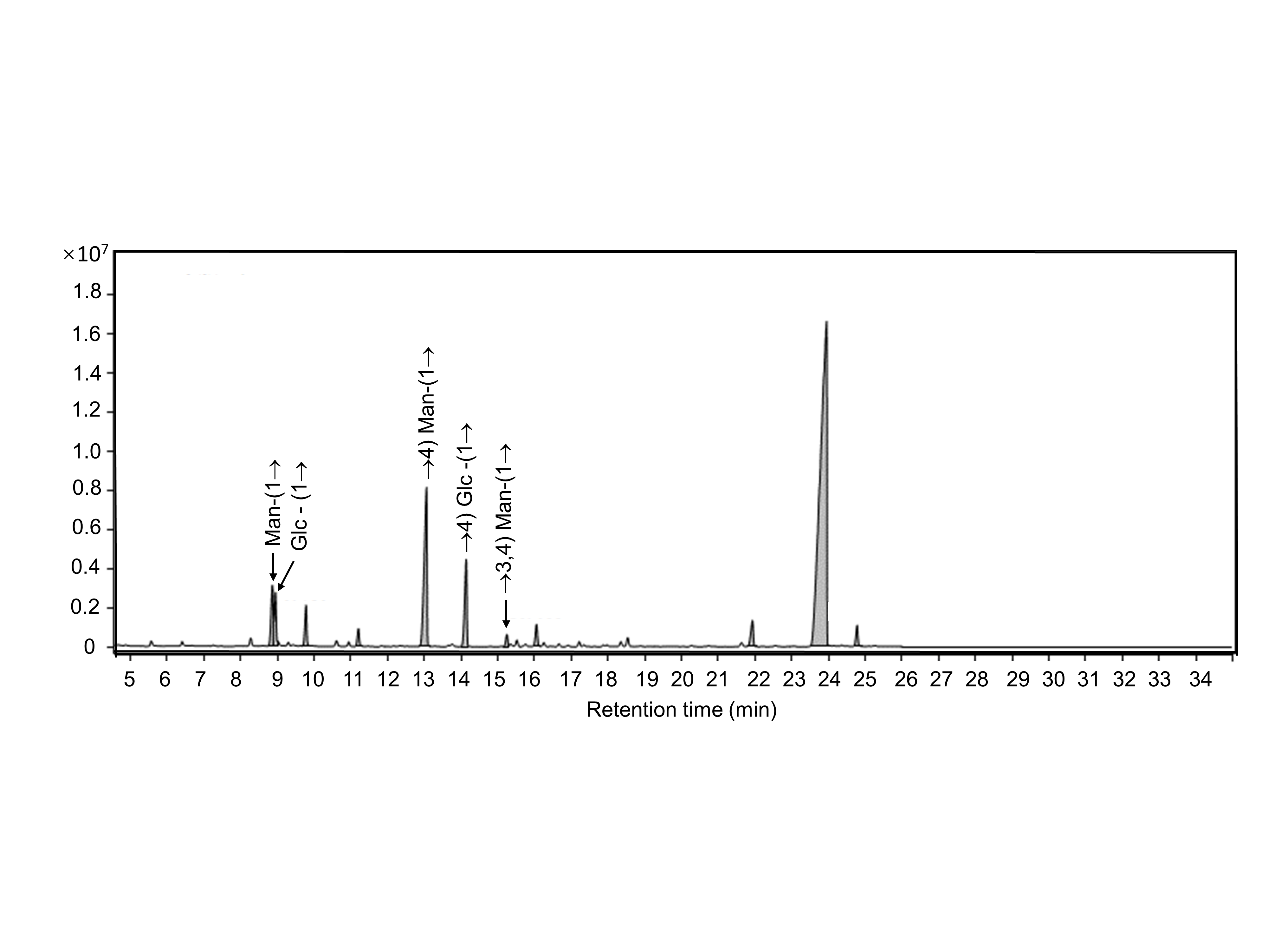


**Fig. S1. The Methylation GC-MS chromatogram of BO.**


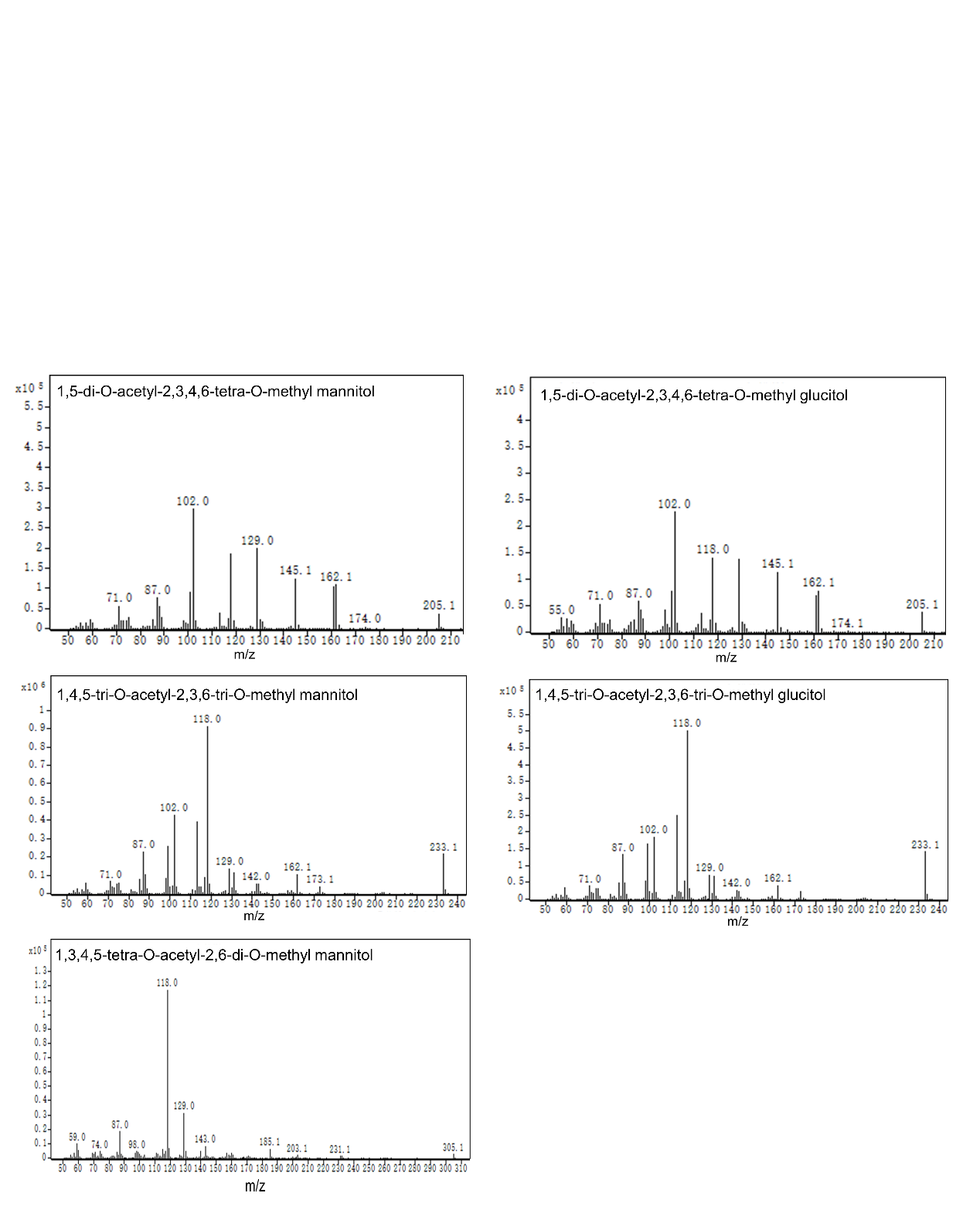


**Fig. S2. Total ion chromatography of BO by GC–MS.**

**
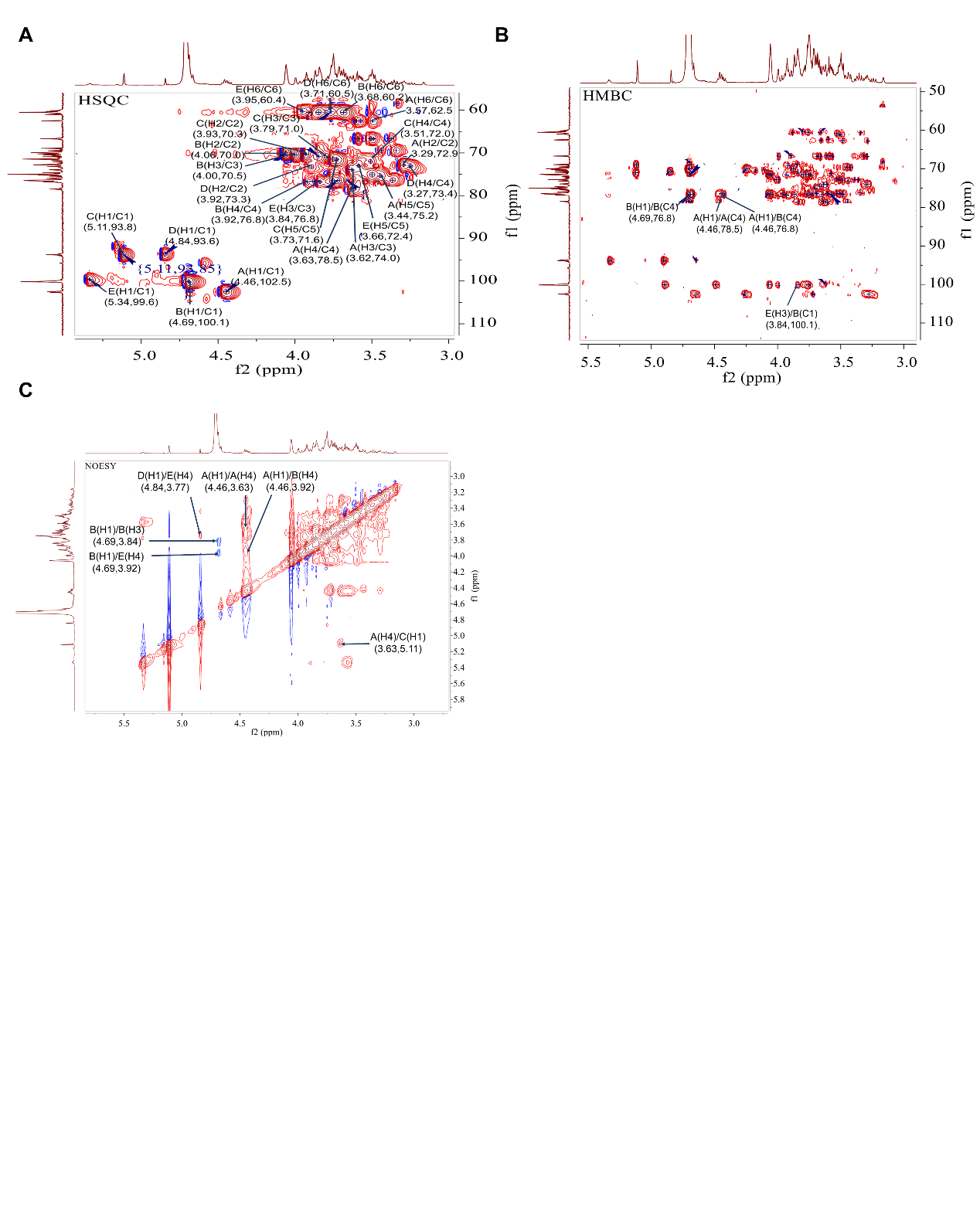
**

**Fig. S3. 2D NMR spectra of BO.** A. 1H–13C HSQC spectrum B. 1H–13C HMBC spectrum; C. 1H-1H NOESY spectrum.


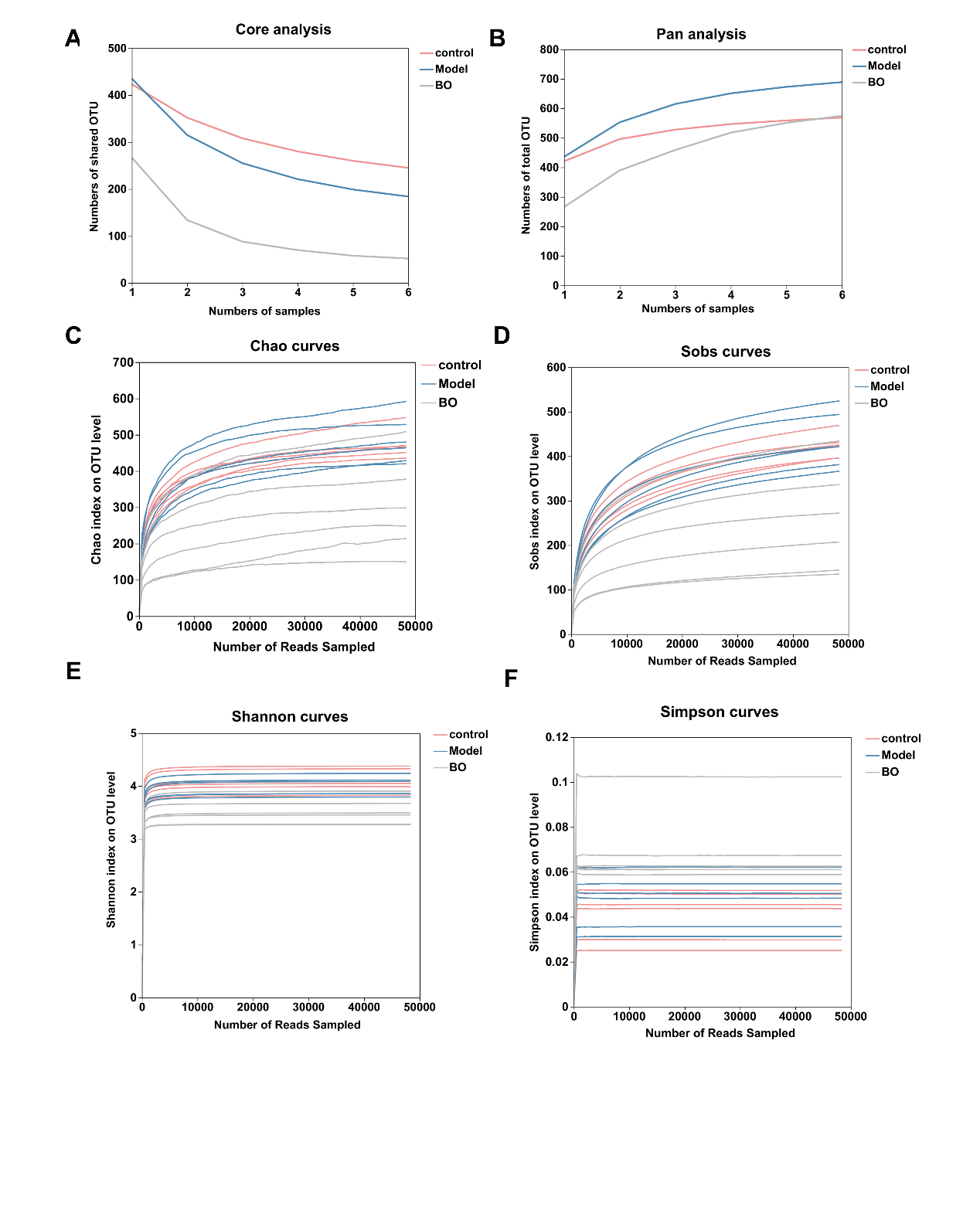


**Fig.S4.** **Pan/Core analysis and Alpha diversity analysis**. A. Core analysis; B. Pan analysis; C-F Alpha diversity (Chao index; Sobs index; Shannon index; Simpson index).


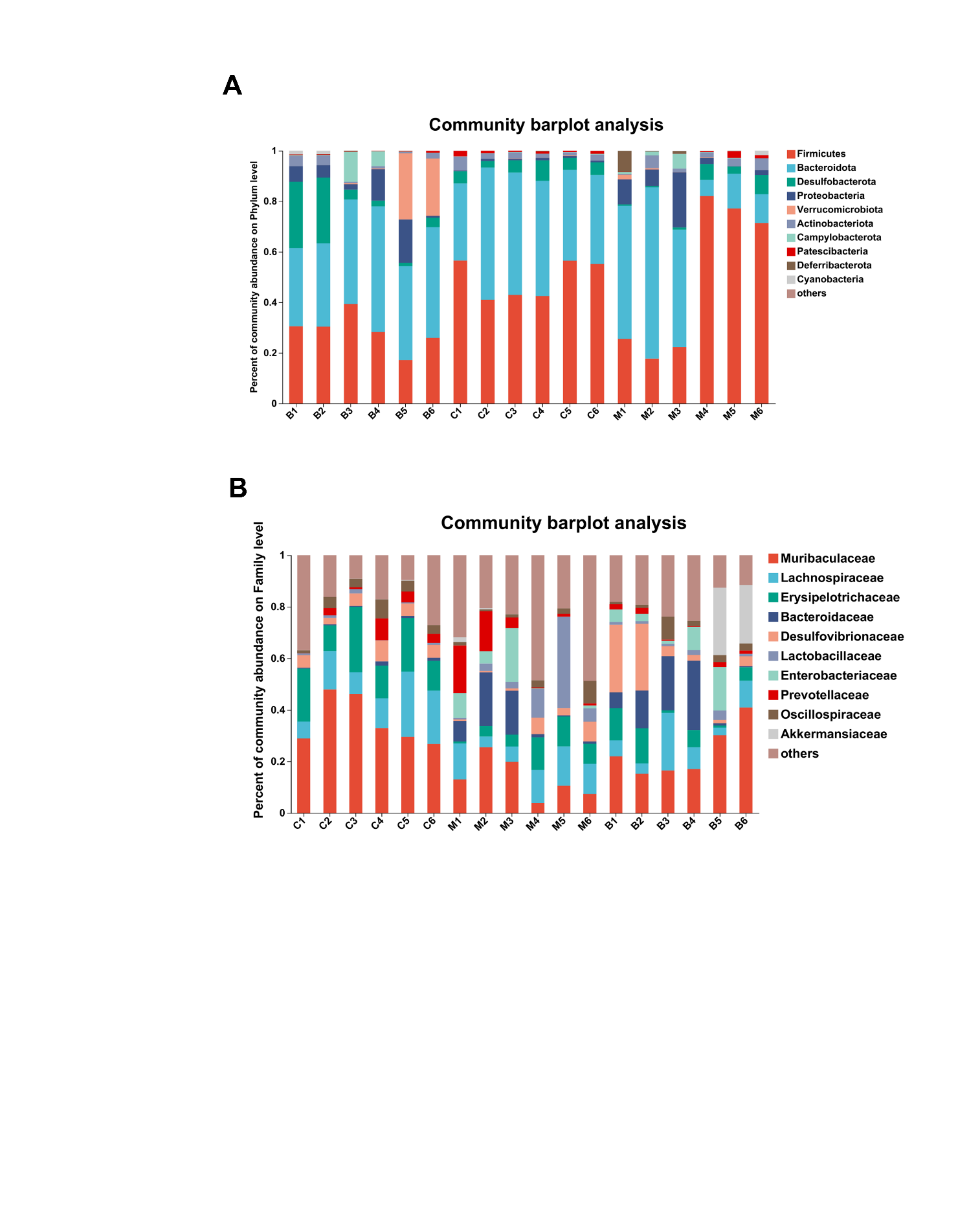


**Fig.S5.** **Species analysis.** A. Community barplot analysis at Phylum level; B. Community barplot analysis at Family level.
